# Selected canine abstracts from the Companion Animal Genetic Health conference 2018 (CAGH 2018): Canine Genetics and Epidemiology: Edinburgh, Scotland. 14-15 May 2018

**DOI:** 10.1186/s40575-018-0062-z

**Published:** 2018-08-29

## Oral presentations

### O1 Glioma in dogs: interest of spontaneous models for the genetics of human gliomas

#### Maud Rimbault^1^, Clotilde De Brito^1^, Aline Primot^1^, Ronan Ulvé^1^, Mélanie Rault^1^, Nadine Botherel^1^, Catherine Escriou^2^, Jérôme Abadie^3^, Laetitia Lagoutte^1^, Delphine Dullin^2^, Dominique Fanuel^3^, Marie-Anne Colle^3^, Patrick Devauchelle^4^, Jean-Laurent Thibaud^4^, Stéphane Blot^5^, Audrey Rousseau^6^, Jean Mosser^1^, Philippe Menei^7^, Benoît Hédan^1^ and Catherine André^1^

##### ^1^Institut de Génétique et Développement de Rennes IGDR, UMR 6290 CNRS/Université de Rennes1, Faculté de Médecine, Rennes, France; ^2^Unité de Pathologie médicale des carnivores (Neurologie et troubles du comportement), VetAgro-Sup, Marcy l’Etoile, France; ^3^Unité AMaROC, Oniris, Atlanpole La Chantrerie, Nantes, France; ^4^MICEN VET, Europarc, Créteil, France; ^5^UPR de Neurobiologie, Ecole Nationale Vétérinaire d’Alfort, Maisons Alfort, France; ^6^Département de Pathologie Cellulaire et Tissulaire, CHU d’Angers, Angers, France; ^7^Département de Neurochirurgie, INSERM U1066, CHU d’Angers, Angers, France

###### **Correspondence:** Maud Rimbault

Human glioma are brain cancers with a dramatic 5 year survival time of 5% even applying the unique reference treatment based on radio- and chemotherapy. Interestingly, among the many dog breeds prone to spontaneously develop cancers, brachycephalic breeds (Boxers, Bulldogs, Boston terriers…) are particularly affected by glial tumors. Dogs share the same environment as humans and have also anatomical and physiological similarities, thus constituting a relevant model for the genetics and therapies of brain tumors.

Thanks to the national Cani-DNA biobank and its veterinary network (the 4 Veterinary Schools, Antagene, private practices and cancer centers) managed at CNRS Rennes (France), samples for 50 glioma affected and >100 control dogs, as well as 1400 brachycephalic dogs have been collected and DNA extracted and stored.

With the goal to compare dog and human gliomas in mind, we performed a retrospective study of 100 canine glioma cases, allowing a clinical, epidemiological and histological characterization of these canine tumors. The predominant localization of glioma to the frontal lobe, predisposed breeds (mainly brachycephalic dogs from the European Mastiff line) and mean age of onset were revealed by the analysis of 20 cases with imaging and 15 cases with histology. We showed that dog gliomas present surprising anatomic and clinical homologies, with comparable histopathological subtypes as in human gliomas.

These results led us to analyze 2 cases for which brain tissue had been collected. We identified a BRAF-MBP gene fusion in one case using RNAseq and we are currently checking for recurrence in the collected samples, as well as for the presence of this translocation in human glioma cases. Using affected cases and controls of the same breeds, we plan to pursue the identification of somatic alterations by transcriptome analyzes (RNAseq) and exome sequencing (WES) and to carry out genetic linkage and/or genetic association studies (GWAS) to identify genomic regions involved in predisposition. We will also search if and how the artificial selection that led to specific morphological characteristics, such as the shape of the dog’s skull (brachycephaly), would have also led to glioma predisposition.

Keywords: Canine, Genomics and Variation, Inherited Disease

### O2 Use of cross-country data for estimation of heritability of longevity and heart-related deaths in Doberman Pinscher

#### Joanna J. Ilska^1^, Paolo Gottardo^2^, John Hickey^1^, Dylan Clements^3^

##### ^1^The Roslin Institute and Royal (Dick) School of Veterinary Studies, Edinburgh, Scotland; ^2^Italian Brown Swiss Breeding Association, Italy; ^3^Royal (Dick) School of Veterinary Studies and The Roslin Institute, Edinburgh, Scotland

###### **Correspondence:** Joanna J. Ilska

Genetic improvement with the use of Estimated Breeding Values (EBV) is a method which, after decades of successful and validated use in livestock species, slowly gains recognition in the world of dogs breeding. However, accurate EBV prediction for complex traits requires large datasets of phenotyped and related animals. While generation of such datasets is possible in the most popular dog breeds, for many other breeds reaching sufficient numbers within any national database is not likely. Further, collection of the data pertaining to diseases through national Kennel Clubs is usually limited to very few already established grading systems for specific diseases, such as British Veterinary Association’s scheme for Hip and Elbow Dysplasia. Thus, databases created by independent breed societies combining records across countries and on breed-specific issues, could become sources of data for the genetic analyses and EBV predictions in numerically small breeds. We present a preliminary analysis of the heritability of longevity and heart-related deaths (HEART) in Doberman Pinscher, based on data collated by The Doberman Welfare Community (DWC) – an independent group of breed enthusiasts. The data included over 350,000 dogs over 37 genera tions, born between 1890 and 2017, and from 18 regions. Phenotypic records on longevity and cause of death were recorded for 10,549 and 5,844 dogs respectively. Neither longevity nor causes of death are currently recorded by national kennel clubs, thus highlighting the role of the DWC in collecting this type of data. Among the causes of death, HEART were most common (48%), and more frequent in males than females (55% males, 45% females). The average longevity (LONG – number of months between birth and death) was 89 months (7 years) for males and 100 months (8 years) for females.

A number of mixed linear models were fitted to identify significant environmental factors affecting LONG and HEART, and to estimate heritability of the traits. LONG was Box-Cox transformed to improve normality of the data, and binomial models were fitted for the heritability estimation of the underlying liability for the HEART. Factors identified as significant for HEART were sex, region, season of birth, and year of death. LONG was affected by year and season of birth, as well as year of death. Heritability of the HEART and LONG was 0.29 ( 0.02) and 0.11 ( 0.02) respectively. To the best of our knowledge, these are the first published estimates of heritability of longevity and heart-related deaths in Dobermans using owner-collated data. A significant genetic variance detected for both traits indicates that selection could bring improvement in these traits, which is particularly important for HEART – heart conditions are believed to affect as many as 20% of Dobermans, and the symptoms of the disease often appear after a dog has already been used for breeding. Further, significant estimates obtained in the presented analyses indicate validity of the data, thus opening a new window of opportunity for genetic analyses of complex traits in numerically small breeds through the recruitment and collation of data by breed enthusiasts.

Keywords: Canine, Genomics and Variation, Inherited Disease

### O3 Canine breed specific cancers as natural models for rare and/or aggressive human cancer types: examples of sarcomas, melanomas, lymphomas and gliomas

#### Benoit Hédan^1^, Mélanie Rault^1^, Ronan Ulvé^1,2^, Aline Primot^1^, Edouard Cadieu^1^, Clotilde de Brito^1^, Nadine Botherel^1^, Maud Rimbault^1^, Jérôme Abadie^3^, Anne-Sophie Guillory^1^, Anais Prouteau^1^, Amaury Vaysse^1^, Patrick Devauchelle^4^, Annabelle Garand^1^, Celine Le Beguec^1^, Laetitia Lagoutte^1^, Valentin Wucher^1^, David Gilot^1^, Thomas Derrien^1^, Christophe Hitte^1^, Catherine André^1^

##### ^1^Institut Génétique et Développement de Rennes, CNRS-UMR6290, University Rennes1, 35000, Rennes, France; ^2^BIOTRIAL Pharmacology, Unite de pharmacologie préclinique, 7-9 rue Jean-Louis Bertrand, 35000, Rennes, France; ^3^ONIRIS, AMaROC, Ecole Nationale Vétérinaire, Agroalimentaire et de l’Alimentation Nantes Atlantique, 44307, Nantes, France; ^4^Centre de Cancérologie Vétérinaire, MICEN Vet 58 rue Auguste Perret, 94000, Creteil, France

###### **Correspondence:** Catherine André

Through the French Cani-DNA biobank, developed in the team since 2005, we have collected over 3000 samples (blood and paired tumour/normal tissues) form many dogs affected by breed specific cancers, as well as controls of the same breeds, for which there are specific issues in the human corresponding cancers. Indeed, naturally occurring canine cancers are recently receiving attention in comparative oncology because of their high similarity to human cancers both in their clinical and histological presentations as well as in their response to treatments.

We have constituted large collections of cases and controls as well as large family pedigrees and through genome wide association studies (GWAS) and genetic linkage approaches, we have identified predisposition loci for Histiocytic Sarcoma (HS) and oral melanomas. In parallel, through the search of somatic genetic alterations in the tumour (whole exome sequence -WES-; capture/sequencing and RNAseq techniques), we have identified relevant genetic alterations in canine lymphomas, sarcomas, melanomas and gliomas. Either we found new genes implicated in dogs and we could identify the same genes in the corresponding human cancers, or we found already known genes, especially oncogenes with the same hotspots than in humans, as well as gene fusions with the same partners and the same over-expression mechanism than in humans, for lymphoma, sarcoma and glioma (Ulvé, Rault et al., 2017). We have identified such genes and their pathogenic somatic alterations for HS (TP53 and a MAPK oncogene), melanomas (over 50 genes, including PTEN, NRAS), lymphomas (26 genes, including cyclins) and gliomas (a BRAF- MBP fusion). For oral melanomas, we also have identified specific Copy Number alterations (CNA) that we showed to be significantly linked to survival.

Finally, we developed cell lines for these canine cancers (8 for HS, 10 for oral and uveal melanomas, 2 for gliomas and 1 for lymphoma), and were able to demonstrate the effect on proliferation, of drugs targeting genes coding for MAPK pathway oncogenes and cyclin genes, for HS and lymphomas respectively. We thus showed that canine cancers might be highly useful for clinical trials, as in vitro and in vivo models to screen drugs in dog/human homologous cancers, prior to test them in humans, in the frame of the treatment of the dogs and with the owner partnership and consent. Finally, to also benefit breeders, we also developed a genetic risk test for Histiocytic Sarcoma, made of 9 markers predictive of a “protective” or “at risk” haplotype and status, available for breeders to help their selection against HS in the Bernese Mountain Dog breed.

To conclude, these genetic findings bring a better understanding of the genetics and potential treatments for dog but also for human medicine. More widely, these results show the interest of the dog model to decipher the genetic bases and plan clinical trials in dogs for rare and/ or aggressive-refractory human cancers.

### O4 Association of primary open angle glaucoma ADAMTS17 mutations with height in two domestic dog breeds

#### Emily Jeanes^1^, James Oliver^2^, Sally L. Ricketts^2^, David Gould^3^, Cathryn Mellersh^2^

##### ^1^Centre for Small Animal Studies, Animal Health Trust, Lanwades Park, Kentford, Newmarket, CB8 7UU, UK; ^2^Canine Genetics Research Group, Kennel Club Genetics Centre, Animal Health Trust, Lanwades Park, Kentford, Newmarket, CB8 7UU, UK; ^3^Davies Veterinary Specialists, Manor Farm Business Park, Higham Gobion, Hitchin, SG5 3HR, UK

###### **Correspondence:** Sally L. Ricketts

There are currently five known ADAMTS17 mutations in the dog that are associated with the development of either primary open angle glaucoma or primary lens luxation. Interestingly, these mutations have been identified in breeds of generally short stature including terriers and Basset breeds. In humans, mutations in the ADAMTS17 gene are associated with Weill-Marchesani syndrome – a disorder whose clinical characteristics include ocular manifestations such as microspherophakia, myopia, glaucoma, and cataract, in addition to brachydactyly and short stature. This led us to hypothesise that these mutations may also be associated with height in these breeds. To test this, we conducted an association analysis between breed-specific ADAMTS17 mutations and height in two of these breeds – the Petit Basset Griffon Vendeen and Shar Pei. Two hundred and twenty-seven Petit Basset Griffon Vendeen and 65 Shar Pei were genotyped for their breed-specific ADAMTS17 mutations. The height of each dog was measured at the withers. We used linear per allele regression to assess the association between ADAMTS17 mutations and height as a continuous variable, and linear regression and log-likelihood ratio tests to assess the shape of the association by comparing a general model with a linear per allele model. The mean heights of affected (n=21), carrier (n=84) and clear (n=122) Petit Basset Griffon Vendeen were 33.41 cm, 34.78 cm and 34.93 cm, respectively. The mean heights of affected (n=9), carrier (n=30) and clear (n=26) Shar Pei were 43.32 cm, 47.93 cm and 48.38 cm, respectively. Each breed-specific ADAMTS17 mutation showed a strong association with height in both breeds: Petit Basset Griffon Vendeen (P=7.9 x 10-3); Shar Pei (P=6.9 x 10-5). The shape of the associations appeared similar between the two breeds. In humans, ADAMTS17 affects skeletal development by modulating the extracellular matrix. A similar mechanism may be present in the dog. We speculate that selection for short stature might have inadvertently increased ADAMTS17 mutant allele frequencies and thus increased prevalence of primary open angle glaucoma in these breeds.

Keywords: Canine, Morphology

### O5 Detection and characterisation of a genetic association with Norwich Terrier Upper Airway Syndrome

#### Thomas Marchant^1^, Elisabeth Dietschi^2^, Ronan Harrington^1^, Michaela Drögemüller^2^, Ulrich Rytz^3^, Elaine A. Ostrander^4^, Tosso Leeb^2^ & Jeffrey J. Schoenebeck^1^

##### ^1^The Royal (Dick) School for Veterinary Studies and Roslin Institute, The University of Edinburgh, Easter Bush, Midlothian, EH25 9RG, UK; ^2^Institute of Genetics, Vetsuisse Faculty, University of Bern, 3001, Bern, Switzerland; ^3^Department of Clinical Veterinary Medicine, Division of Small Animal Surgery, Vetsuisse Faculty, University of Bern, 3001, Bern, Switzerland; ^4^Cancer Genetics Branch, National Human Genome Research Institute, Bethesda, MD, USA

###### **Correspondence:** Thomas Marchant

In domestic dogs, the “flat-faced” brachycephalic head shape is a risk factor for developing the respiratory defect, Brachycephalic Obstructive Airway Syndrome (BOAS). As the popularity of breeds such as the French bulldog continues to increase in the UK, so too are the expected incidences of BOAS. For this reason, we became interested in the Norwich terrier, a non- brachycephalic breed which presents with Upper Airway Syndrome (UAS), a condition highly reminiscent of BOAS. Here, we have studied this single breed to identify genetic association(s) with UAS. Pathological assessments and grading from laryngoscopic examinations held at the Vetsuisse Faculty of the University of Bern, were used as phenotypes in conjunction with microarray genotypes to perform GWAS. In total, 233 Norwich terriers were examined. We identified the same QTL on canine chromosome (CFA) 13 to be associated with the abnormal positioning of laryngeal cartilage and everted saccules in the dogs most severely affected by UAS. We phased genotypes at the CFA13 QTL to conduct haplotype mapping, which led us to define a 413 kb critical interval which encompasses a single positional candidate gene. The derived haplotype within this interval is overrepresented: it is found to be homozygous in 61 of 81 (74%) severely affected cases. In contrast, this homozygous haplotype was identified among 7 of 86 (8.1%) mild/unaffected controls. We have resequenced four dogs representing phenotypic extremes to sixteen-fold depth to identify putatively causal variants. We will provide an update to this ongoing project, which is expected to guide Norwich terrier breeding and inspire additional exploration of the CFA13 locus to improve animal welfare.

Keywords: Canine, Inherited Disease, Morphology

### O6 Identification and characterisation of a mutation associated with cerebellar ataxia in the Norwegian Buhund dog breed

#### Chris Jenkins^1^, Lajos Kalmar^3^, Lorenzo Mari^2^, Ellen Schofield^1^, Cathryn Mellersh^1^, Luisa De Risio^2*^, Sally Ricketts^1*^

##### ^1^Kennel Club Genetics Centre, Animal Health Trust, Newmarket, UK; ^2^Neurology/ Neurosurgery Service, Centre for Small Animal Studies, Animal Health Trust, Newmarket, UK; ^3^Department of Veterinary Medicine, University of Cambridge, Cambridge, UK

###### **Correspondence:** Chris Jenkins

*Authors contributed equally

Inherited ataxias are typically incurable and lack disease-modifying treatments. Four Norwegian Buhunds were diagnosed with cerebellar ataxia at the Animal Health Trust. Pedigree analysis was suggestive of an autosomal recessive mode of inheritance, which is typical of inherited canine ataxias. The causal variant for ataxia in these dogs was hypothesised to be private to the breed. Whole genome sequence (WGS) was obtained for two sibling cases, which were compared to WGS from 405 dogs of other breeds. The WGS used included 44 which were generated for the study of other diseases in our laboratory, and 361 additional WGS which are part of the Dog Biomedical Variant Database Consortium. Filtering out benign variants left nine that were present only in the cases and predicted to directly affect a protein coding sequence or alter a transcript. These were assessed in 14 related and unrelated Buhunds, leaving one variant that fully segregated with the disease. Its association with ataxia was confirmed by typing in an extended set of 148 Buhunds containing two additional cases, and its absence in 359 dogs of 122 other breeds. This research has resulted in the development of a DNA test enabling breeders to avoid producing affected dogs. Importantly, the causal mutation is within a gene not previously reported to be associated with ataxia in any species. A combination of approaches was used to characterise this gene in the dog, as the current CanFam 3.1 annotation is incorrect. The gene is highly conserved and, in humans and mice, encodes multiple transcripts with alternative first exons. Expression of multiple transcripts in the canine cerebellum was confirmed through RNA sequencing, and through RT-PCR of samples from two Norwegian Buhund cases and five unaffected dogs of other breeds. RT-qPCR analysis and in-silico protein modelling have been used to further investigate the mutation’s effect on RNA expression and protein stability.

Keywords: Canine, Inherited Disease

### O7 Ontogenic transcriptomic profiling identifies signalling pathways driving pathogenesis in canine myxomatous mitral valve disease

#### Greg R. Markby^1^, Kim M. Summers^1,2^, Vicky E. MacRae^1^, Brendan M. Corcoran^1,3^

##### ^1^The Roslin Institute, Edinburgh, Scotland; ^2^Mater Research Institute, Brisbane, Australia; ^3^The Royal (Dick) School of Veterinary Studies, Edinburgh, Scotland

###### **Correspondence:** Brendan M. Corcoran

Chronic degenerative diseases (CDGs) are a major welfare concern in canine medicine with myxomatous mitral valve disease (MMVD) being an important example. For some breeds CDGs can have an inherited basis, but often this is a polygenic trait and so understanding the mechanisms that drive disease pathogenesis requires examining molecular events in tissue. Specifically for CDGs this requires examination in both temporal and spatial terms changes in gene and protein expression. In this study we have examined the valvular gene expression at different stages of disease (temporal), different locations (spatial) and in different cell culture models of MMVD.

Methods

Transcriptomic profiling (Affymetrix canine 1.1ST microarray), with validation using RT-qPCR for selected genes, was performed on, whole valves from normal and the 4 grades of MMVD (n=6),normal and diseased regions of grade 2 valves (n=7), and cultured (all experiments n=3) normal and diseased valve interstitial cells (VICs), normal cells treated with 5ng/μL TGFβ1 and diseased cells treated with 10μM of the TGFβ pathway inhibitor SB431542. Microarray data were analysed using a range of bioinformatics platforms (Affymetrix Console, IPA, Miru (Biolayout Express)).

Results

Significantly differentially expressed genes (DEG) were identified comparing: 1) normal and the 4 grades of MMVD (1002 genes); 2) diseased and normal tissue within the same valve (315 genes); 3) normal and diseased VICs (1027 genes); 4) normal VICs and normal VICs treated with TGF-β1 (302 genes); 5) diseased VICs and diseased treated with VICs SB431542 (269 genes).

Grade-dependent up and down regulated gene clusters were identified, and microarray data were validated by RT-PCR for ACTA2, TAGLN and 5HTR2B. Important GO-terms were found to be associated with myofibroblast differentiation and extracellular matrix homeostasis. In all data sets altered DEGs implicated TGF-β1 as the important up-stream regulator of disease pathogenesis, with minor contributions from TNF and IFNG. 75 DEGs were shared in common between grade 4 whole valve and the diseased sections of the dissected valves. Cultured cell data, in addition to TGFβ1, predicted genes involved in cell cycle and apoptosis as important up-stream regulators.

Conclusions

This study shows how transcriptomic profiling of chronic degenerative disease over an entire lifetime, in tandem with cell culture models, can identify the signalling pathways important in disease pathogenesis. TGFβ1 signalling has been identified as the fundamentally important pathway in MMVD initiation and development, and progression to eventual end-stage valve pathology.

The authors gratefully acknowledge funding from Dogs Trust

Keywords: Canine

### O8 Genetic analyses of Lhasa Apso dogs with progressive retinal atrophy identifies a LINE-1 insertion in the promoter region of a retinal candidate gene

#### Rebekkah J. Hitti^1,2^, Louise M Burmeister^1^, Sally L Ricketts^1^, Louise Pettitt^1^, Mike Boursnell^1^, Ellen C Schofield^1^, David Sargan^2^, Cathryn Mellersh^1^

##### ^1^Kennel Club Genetics Centre, Animal Health Trust, Lanwades Park, Newmarket, Suffolk, CB8 7UU, UK; ^2^Department of Veterinary Medicine, University of Cambridge, Madingley Road, Cambridge, CB3 0ES, UK

###### **Correspondence:** Rebekkah J. Hitti

Canine progressive retinal atrophy (PRA) is a degenerative retinal disease characterised by photoreceptor degeneration over time, increasing in severity and ultimately leading to vision loss. PRA affects multiple breeds and significantly impacts welfare. In the Lhasa Apso (LA) dog, PRA manifests typically as a mid-late onset form. Utilisation of whole-exome sequencing (WES) data previously generated in our laboratory from three PRA-affected LA (cases) and three PRA-unaffected LA (controls) did not reveal any obvious exonic or splice site polymorphisms segregating with the disease, indicating a non–coding mutation. This presented the opportunity for further investigations using a genome-wide association study (GWAS) and whole-genome sequencing (WGS) approach to identify the genetic cause of PRA and develop a DNA test.

A GWAS was conducted by genotyping 44 LA dogs (17 cases, 27 controls) on the Illumina Canine HD 170K chip. Allelic association statistics were adjusted for multiple testing using the PLINK Max(T) permutation procedure, and for population stratification and relatedness using Efficient Mixed-Model Association eXpedited (EMMAX). After stringent filtering and quality control, we tested 108,263 SNPs on 42 dogs, comprising 15 cases and 27 controls (call rate ≥97%; minor allele frequency ≥95%; genotype calls ≥90%). Analysis revealed a genome-wide significant association on canine chromosome 33 (-log praw = 2.2 x 10-16) which remained significant after correcting for multiple testing (pgenome = 0.9 x 10-5) and population substructure (p=raw1.6 x 10-17). A 1.3 megabase homozygous disease-associated region was defined, harbouring two candidate genes previously associated with human retinal degeneration.

WGS was undertaken on a single PRA affected LA, and manual interrogation of the critical region in identified a long interspersed nuclear element-1 (LINE-1) insertion, situated within the predicted promotor region of a retinal candidate gene. Due to the position of the LINE-1 insertion, it was not detected in the original WES data of the same case. The LINE-1 insertion was genotyped in 447 dogs across 122 breeds, including 63 LA dogs, and is private to the LA. Seventeen LA dogs (all clinically affected with PRA) were homozygous for the LINE-1 insertions, eight were heterozygous and thirty-eight were homozygous for the wildtype allele.

As a result of this study a DNA test for this form of PRA, termed PRA4, has been developed at the Animal Health Trust. To date, 457 LA from 15 countries have been tested for PRA4 (354 UK dogs; carrier frequency 17%; allele frequency 0.9054). This study highlights the power of utilising several genetic approaches to identify a PRA mutation and develop a diagnostic test to help dog breeders make informed breeding choices, minimising the risk of producing PRA-affected LA dogs.

Keywords: Canine, Inherited Disease

### O9 A dog spontaneous model for human sensory neuropathies: identification of a mutation in a regulatory region of GDNF and DNA screening in human patients

#### Solenne Correard^1*^, Jocelyn Plassais^1*^, Laëtitia Lagoutte^1^, Manon Paradis ^2^, Nadine Botherel^1^, Benoit Hédan ^1^, Eric Guaguère^3^, Ines Mademan^4^, Agnes Méreau^5^, Anne-Sophie Lia^6^, Christophe Hitte^1^, Nathalie Bonello-Palot^7^, Pascale Quignon^1^, Sylvie Odent^8^, Yline Capri^9^, Julien Cassereau^10^, Vincent Timmerman^11^, Valerie Delague^12^, Emmanuelle Bourrat^13^, Dominique Bonneau^14^, Jean-Michel Vallat^15^, Eric Leguern^16^, Thomas Derrien^1^, Catherine André^1^

##### ^1^Dog genetics team - IGDR, UMR6290/CNRS/Université de Rennes 1, Rennes, France; ^2^Department of Clinical Sciences - Faculté de Médecine Vétérinaire, University of Montreal, St-Hyacinthe, Canada; ^3^Veterinary clinic Saint Bernard, Lomme, France; ^4^Neuro- genetics Group - Center for Molecular Neurology – VIB, Antwerpen, 2610, Belgium; ^5^Gene expression and development - IGDR, UMR6290/CNRS/Université de Rennes 1, Rennes, France; ^6^Laboratoire de Biochimie et Génétique Moléculaire - CHU Dupuytren, Limoges, France; ^7^Laboratoire de génétique moléculaire - Centre de référence maladies rares Thalassémies - Hôpital de la Timone - AP-HM, Marseille, France; ^8^Centre Hospitalier Universitaire de Rennes - Service de Génétique Clinique, Rennes, France; ^9^Department of Genetics - hôpital Robert-Debré AP-HP, Paris, France; ^10^Centre de Référence Maladies Neuromusculaires de l’Enfant et de l’Adulte Nantes-Angers - Centre Hospitalier Universitaire d’Angers, Angers, France; ^11^Peripheral Neuropathy Research Group - Institute Born Bunge - University of Antwerp, Antwerpen, Belgium; ^12^Inserm - UMR_S 910 - Aix Marseille Université – GMGF, Marseille, France; ^13^Service de Dermatologie - AP-HP - Université Paris VII Sorbonne Paris Cité - Hôpital Saint-Louis, Paris, France; ^14^Service de génétique - plateau de biologie hospitalière - CHU d’Angers, Angers, cedex 09, France; ^15^Department of Neurology - National Reference Center for Rare Peripheral Neuropathies, University Hospital, Limoges, France; ^16^INSERM - U1127 – ICM – CNRS - UMR 7225 - Sorbonne Universités – UPMC - Department of Genetics - Pitié-Salpêtrière Hospital - Public Hospital Network of Paris, Paris, France

###### **Correspondence:** Solenne Correard

*Authors contributed equally

In humans, there are many forms of sensory neuropathies, associated or not with a loss of sensitivity to pain and sometimes accompanied by self-mutilation. Although to date 13 genes have already been implicated in this disease, they do not explain the genetic causes of all patients.

Similar neuropathies are diagnosed in dogs and several breeds are at risk to develop certain forms. Neuropathy has been described in hunting dogs, where the condition results in progressive mutilation of the distal extremities of the paws (Paradis et al., 2005). Pedigree analysis led to conclude to a monogenic autosomal recessive mode of inheritance. Blood samples from affected and unaffected hunting dogs from France and from Canada were collected through the French Cani- DNA biobank (dog-enetics.genouest.org). Genetic studies (GWAS and sequencing) led to the identification of a locus on canine chromosome 4, and to a mutation located 90kb upstream GDNF, a gene encoding a neurotrophic factor involved in the survival of dopaminergic neurons. This mutation segregates as expected in 300 hunting dogs of known clinical status and is not found in 900 dogs of 90 other non-predisposed breeds. Functional experiments have shown that the mutation causes a decrease of GDNF expression in the dorsal root ganglia and also a decrease in the affinity of a regulatory complex for the DNA sequence to which it binds (Plassais et al., 2016). This gene had not previously been involved in human forms of sensory neuropathy and appears a good candidate. Through French and Belgium reference centers, we collected 111 DNAs of patients affected with different forms of sensory neuropathies and we sequenced GDNF exons as well as two regions predicted as regulatory, orthologous to the mutated regulatory region in the dog.

23 variants were identified and classified:

i. New variants (not listed in human databases).
ii. Rare variants (listed in databases with a minor allele frequency inferior to 1%).

No new variants have been found in the coding parts of the gene, however, 6 new variants have been identified in the UTRs and regulatory regions upstream GDNF and 17 rare variants were also identified.

In conclusion, the dog model has allowed to identify a new gene for canine and potentially human sensory neuropathies. New and rare variants in this gene are being analyzed to tentatively identify their potential role in human neuropathies.

References

Plassais et al. 2016. A point mutation in a lincRNA upstream of GDNF is associated with a canine insensitivity to pain: a spontaneous model for human sensory neuropathies. Plos Genetics 2016

Keywords Canine, Genomics and Variation, Inherited Disease

### O10 Extensive whole genome sequencing comparisons in dogs elucidates a putative novel candidate gene for retinal degeneration

#### Rebekkah J. Hitti^1,2^, James A. Oliver^1^, Ellen C. Schofield^1^, Anina Bauer^3^, Tosso Leeb^3^, David Sargan^2^, Cathryn S. Mellersh^1^

##### ^1^Kennel Club Genetics Centre, Animal Health Trust, Lanwades Park, Newmarket, Suffolk, CB8 7UU, UK; ^2^Department of Veterinary Medicine, University of Cambridge, Cambridge, UK; ^3^Institute of Genetics, University of Bern, 3001, Bern, Switzerland

###### **Correspondence:** Rebekkah J. Hitti

Retinitis pigmentosas (RP) are genetically heterogeneous, progressive diseases characterised by retinal degeneration and causing loss of vision before middle age, and affecting in 1 in 2000 humans. The canine equivalent, progressive retinal atrophy (PRA) is untreatable and affects multiple dog breeds, significantly impacting dog welfare.

A novel form of PRA was diagnosed in a family of Giant Schnauzer dogs, where three out of seven littermates presented with clinical signs of PRA around four years of age. The sire and dam were clinically unaffected and therefore considered likely to be obligate carriers of an autosomal recessive mutation causing PRA in this family. We sought to identify the causal mutation of PRA in this Giant Schnauzer family with the ultimate aim of developing a DNA test, a tool which breeders could utilise to prevent this form of PRA becoming widespread in the breed.

Whole genome sequencing (WGS) of two PRA affected full-siblings and their unaffected parents was performed. Variants were filtered based on those segregating appropriately for an autosomal recessive disorder and those with a predicted pathogenic effect on the coding sequence and protein. Successive filtering against a total of 568 genomes (including genomes from the Dog Biomedical Variant Database Consortium and the Animal Health Trust Give a Dog a Genome bank) reduced an initial set of > 20 million variants down to a single candidate variant in a novel gene not previously associated with retinal degeneration in any species.

The candidate variant was genotyped in a total of 1,444 dogs of 175 breeds, 10 cross breed dogs and 3 wolves, with our three PRA-affected Giant Schnauzers being the only homozygotes identified to date. Nine Giant Schnauzer heterozygotes were identified in addition to heterozygotes in three additional breeds of German origin, including the German Giant (Gross) and Medium (Mittel) Spitz and Miniature Longhaired Dachshund (MLHD). The genotyping of German Spitz varieties, including 110 Giant Spitz, 21 Medium Spitz, 24 Miniature (Klein) Spitz, 17 Pomeranian (Zwerg) Spitz, and an additional 27 Giant Schnauzers was carried out by collaborators at the University of Bern. We screened a total of 163 MLHD for the candidate variant. Seven German Giant Spitz, one German Medium Spitz and six MLHD heterozygotes were identified, suggesting this may be an ancestral, but rare, mutation.

This study highlights the power of using WGS to identify novel genes associated with disease using a very small number of cases. This novel candidate gene, harbouring a variant that is predicted to be the causal mutation of PRA in the Giant Schnauzer, could provide insights into gene discoveries in human retinal degenerations. Further functional study options are being explored to confirm the candidate gene’s role in retinal function and maintenance.

Keywords: Canine, Inherited Disease

## Poster presentations

### P1 Swedish actions to “handle” Brachycephalic Obstructive Airway Syndrome (BOAS) related health issues

#### Åke Hedhammar^1,2^, Linda Andersson^2^, Sofia Malm^2^, Helena Skarp^2^, Anne-Sofie Lagerstedt^1^

##### ^1^Department of Clinical Sciences, Swedish University of Agricultural Sciences, Uppsala, Sweden; ^2^The Swedish Kennel Club, Box 771, SE-19127, Sollentuna, Sweden

###### Correspondence: Åke Hedhammar

The poster is reviewing Swedish actions to improve health issues related to Brachycephalic Obstructive Airway Syndrome (BOAS) and handling the increased attention and awareness of the syndrome.

These actions are

1. forming of working groups composed of various stakeholders, arranging conferences and production of educational material for breeders, judges and veterinarians (a,b).
2. reviewing the epidemiology of BOAS in Swedish dogs by breed club health surveys and insurance data and focusing on a published report in Swedish (Lagerstedt et. al 2018) on 300 dogs operated at 12 clinics in Sweden during 2014-15-16 (including information on breed, gender, age, procedures performed and outcome) Improved diagnostic criteria and follow ups are proposed.
3. initiating a Nordic inventory on the phenotypic and genotypic variation in four brachycephalic breeds – English bulldogs, French bulldogs, Pugs and Boston terriers
  Aim
  To investigate if there is sufficient phenotypic and genotypic variation in four brachycephalic breeds to allow selection for a change in anatomy and thereby reduce the predisposition for Brachycephalic Obstructive Airway Syndrome (BOAS)
  The project is based on “Breed” -gatherings – for dogs of various background arranged for by the breed clubs and supported by the four Nordic Kennel Clubs
  Material
  Data collection by breed on demography: gender, age, country of origin, measures: of weight, BodyConditionScore (BCS) and conformational measurements (i.e. width of Nares (WN), craniofacial ratio (CFR), neck girth ratio (NGR), photo in standardised position (whole body and skull)and surveying general and specific health conditions by a survey to owners and a veterinary examination (clinical data including BOAS)
  Cheek swabs will be collected from each dog for genomic analyses
  Analyses of data is intended to compare variation within and between breeds regarding age, gender, origin and the indicated measures
  Out of all dogs described and sampled, 100 individuals of each breed from each country will be selected for more extensive studies across-breed quantitative trait locus. By performing genotyping using the Illumina high density 170K SNP array of around 400 brachycephalic dogs we will estimate the genomic variation in each breed.
4. Control measures by a reporting form where deaths and performed surgical procedures regarding BOAS are registered as well as an obligatory puppy health certificate to be issued at time of delivery to a new home.
5. The development of a screening procedure for evaluation of breathing capacity , thermoregulation and anatomical features relevant for breathing in adult dogs to initially be used and registered voluntarily but intended to serve in the future as a mandatory request for breeding animals.
6. international collaborations on these issues with Nordic Kennel Clubs and the veterinary organisations (WSAVA / FECAVA) are described as well as Swedish involvement in the BOAS activities within International partnership for dogs( IPFD) and DogWellNet (c,d,e)

**References**

Lagerstedt A-S and Hedhammar, 2018. Surgical procedures performed in Sweden on brachyce- phalic dogs with BOAS. Svensk Veterinärtidning 70:3, 23-27 (in Swedish)

https://www.skk.se/globalassets/dokument/utstallning/special-breed-specific-instructions-a8.pdf

https://www.youtube.com/watch?v=kQ_3f4bLkME&feature=share

https://www.skk.se/globalassets/nku-en/documents/brachyreport.pdf

https://dogwellnet.com/content/hot-topics/brachycephalics/

https://www.fecava.org/files/ckfinder/files/2018_06_Extreme_breeding_adopted.pdf

### P2 Cani-DNA: a French National biobank of canine samples for dog/human compared biomedical research

#### Nadine Botherel^1,2*^, Laëtitia Lagoutte^1,2*^, Benoit Hedan^1,2^, Gilles Chaudieu^3^, Florent Rollin^1,2^, Patrick Devauchelle^4^, Clotilde de Brito^1,2^, Philippe Pilorge^5^, Anne-Sophie Guillory^1,2^, Stéphanie Robin^1,2^, Jérome Abadie^6^, Laurent Tiret^7^, Rachel Lavoue^8^, Marie Abitbol^9^, Anne Thomas^10^, Catherine André^1,2^

##### ^1^CNRS, UMR6290, Institut de Génétique et Développement de Rennes, Rennes, France; ^2^Université de Rennes 1, UEB, Biosit, Faculté de Médecine, Rennes, France; ^3^Clinique vétérinaire Beaulieu, Chamalières, France; ^4^MICEN-Vet, Créteil, France; ^5^Clinique vétéri- naire St Cyr, Rennes, France; ^6^Ecole Nationale Vétérinaire de Nantes, ONIRIS, Nantes, France; ^7^Ecole Nationale Vétérinaire d’Alfort, ENVA, Maisons-Alfort, France; ^8^Ecole Nationale Vétéri- naire de Toulouse, ENVT, Toulouse, France; ^9^Ecole Nationale Vétérinaire de Lyon, VetAgroSup, Marcy l’Etoile, France; ^10^Antagene, Laboratoire de Génétique Animale, La Tour de Salvagny, France; ^11^AFVAC: Association Française des Vétérinaires pour Animaux de Compagnie, Paris, France

###### **Correspondence:** Catherine André

*Authors contributed equally

Dog represents a spontaneous model of many genetic diseases including cancers and other multifactorial diseases as well as monogenic diseases. The last 10 years, genetic tools dedicated to dogs allowed to identify the genetic causes or predisposition to canine and human homologous genetic conditions. More recently, the idea emerged that dogs can be of help for therapeutic trials, to screen and validate new drugs in homologous human cancer types. To this aim, we created the canine Cani-DNA biobank in 2003 to collect blood and tissue samples from dogs affected with genetic diseases and healthy dogs. Cani-DNA contains the native samples and extracted nucleic acids as well as clinical and genealogical data, to distribute samples with high quality controlled procedures. This resource is implemented by a French veterinarian network based on vet practic- es, specialized vet centres and histopathology laboratories. In 2012, a contract signed between Cani-DNA and the 4 French vet schools and the Antagene Company allowed a national organization and international visibility. Cani-DNA joined the French consortium of domesticated animals, CRB-Anim (funded by French government 2012-2020), aiming to combine genetic and reproductive resources. To date, Cani-DNA, with its primary site at CNRS Rennes and secondary sites at the fourth Vet Schools and Antagene contains almost 20 000 DNA extracted from blood and 3000 nucleic acids (DNA, RNA) extracted from tissue samples (tumoral and controls), representing 300 breeds and over 100 genetic diseases. Samples can be sent to implement Cani-DNA and can be distributed for research purposes, upon e-mail ordering (cani-dna@univ-rennes1.fr) or through : http://dog-genetics.genouest.org. We plan to facilitate exchanges and access to such samples for biomedical research with other European biobanks such as those in Bern with 40 000 samples from ~100 breeds (T. Leeb) and Helsinki with 60 000 samples from 330 breeds. (H. Lohi).

Keywords: Canine, Genomics and Variation, Inherited Disease

### P3 Give a Dog a Genome: generating a stake-holder funded bank of whole-genome sequences with which to elucidate benign and disease-associated variation within the canine genome

#### Louise Burmeister, Ellen Schofield, Rebekkah Hitti, Chris Jenkins, Bryan McLaughlin, James Oliver, Louise Pettitt, Sally Ricketts, Cathryn Mellersh

##### Kennel Club Genetics Centre, Animal Health Trust, UK

###### **Correspondence:** Louise Burmeister

The advent of whole genome sequencing (WGS) has promised to revolutionise genetic research, and the rapid fall in per-sample costs in recent years has made the revolution an affordable reality for geneticists. The technology is especially useful for the study of simple Mendelian conditions where disease-causing mutations have the potential to be identified from the WGS of a single case. However, when comparing a typical canine genome with the reference sequence (Can- Fam3.1) or a control genome, at least 2-3 million variants will typically be identified. Many of these variants are likely common polymorphisms which could be excluded by comparing with multiple control genomes. We devised the Give a Dog a Genome (GDG) project to build a resource of canine genetic variants across the genome using WGS; currently projected to contain 90 genomes from 78 breeds, and investigate genetic diseases in at least 69 breeds. We used a crowd-funding approach, with the costs of the project being shared between multiple stakeholders. To date (two years after GDG was launched), 74 samples from 69 breeds have been sequenced comprising 62 dogs affected with a suspected genetic condition (27 conditions in total) and 12 apparently healthy older dogs. The GDG variant bank has been used to validate several disease-associated mutations and DNA tests have been developed to improve the health and wellbeing of dogs. All of the WGS data generated through GDG will be shared with the Dog Biomedical Variant Database Consortium (DBVDC), and specific sequences will be shared with at least 20 scientists from Europe and the USA to contribute to their research.

Keywords: Canine, Genomics and Variation, Inherited Disease

### P4 Population structure of Leonberger dogs

#### Anna Letko^1^, Katie Minor^2^, Jim Mickelson^2^, Franz Seefried^3^, Cord Drögemüller^1^

##### ^1^Institute of Genetics, University of Bern, Switzerland; ^2^Department of Veterinary and Biomedical Sciences, University of Minnesota, Minneapolis, USA; ^3^Qualitas AG, Zug, Switzerland

###### **Correspondence:** Anna Letko

The Leonberger is a giant dog breed formed in the 1850s in Germany. This breed appears to have higher predisposition to neurodegenerative disorders and osteosarcoma than other breeds. Every second polyneuropathy-diagnosed Leonberger can be explained by dominantly inherited ARHGEF10 or GJA9 variants and a recently described recessive NAPEPLD variant identifies a juvenile-onset leukoencephalomyelopathy. Breeders also report shorter lifespan and lower fertility in Leonberger dogs. These problems, combined, imply inbreeding depression. We assessed the genetic diversity of the Leonberger population from extensive pedigree data (including more than 145,000 animals) as well as single nucleotide polymorphism (SNP) genotypes based on 170K array data of 1,175 dogs.

Pedigree analysis was done using open source software EVA v3.0. The completeness index over 5 generations of available pedigrees was above 99% for animals from the latest cohorts and exceeded 80% in 1935. We identified 22 founder animals in the population and a severe bottleneck during the 1940s with only 17 inbred dogs registered in 1946. Since the year 2000 approximately 4,400 dogs are born every year worldwide. The average litter size across cohorts was 6.5 puppies and a constant generation interval of 4 years was observed. The average inbreeding coefficient F was estimated to be 0.29 with a maxF of 0.60. The popular sire effect is apparent since a quarter of all sires produces two thirds of all offspring and the three top males sired more than 330 registered animals each. Additionally, SNP array data of 1,175 individuals sampled worldwide were investigated. These animals represent the current population of Leonberger well and therefore subpopulations were expected due to large geographic distances between breeders. However, multidimensional scaling (MDS) of pairwise genetic distances was carried out and revealed no significant clustering. Additionally, a high level of homozygosity was observed.

Despite increasing population size observed in last cohorts, considerable genetic diversity has been lost due to the bottleneck in the last century. The use of popular sires and high level of inbreeding may have facilitated undesirable genetic traits to spread rapidly within the gene pool of the Leonberger population. Maintaining the genetic diversity is possible through informed selection decisions (especially to include more animals in breeding practice, avoid the use of popular sires and aim to minimize inbreeding) which would contribute to reduce the incidence of health problems. Crossbreeding with several candidate breeds could help optimize long-term genetic diversity.

Keywords: Breed Composition, Canine, Inherited Disease

### P5 Integrated genomic and transcriptomic analyses of long non-coding RNAs in dog as a model of human melanoma

#### Céline Le Béguec^1^, Valentin Wucher^1,2^, Aline Primot^1^, Edouard Cadieu^1^, Lætitia Lagoutte^1,3^, Nadine Botherel^1^, Benoît Hédan^1^, Clotilde De Brito^1^, Anne-Sophie Guillory^1^, Jérôme Abadie^4,5^, Kerstin Lindblad-Toh^6,7^, Catherine André^1^, Thomas Derrien^1^, Christophe Hitte^1^

##### ^1^Univ Rennes, CNRS, IGDR (Institut de génétique et développement de Rennes), UMR 6290, F-35000, Rennes, France; ^2^Present adresse: Centre for Genomic Regulation (CRG), The Barcelona Institute of Science and Technology, Dr. Aiguader 88, Barcelona, 08003, Spain; ^3^Present adresse: UMR PEGASE, Agrocampus Ouest, INRA, 35042, Rennes, France; ^4^Laboratoire d’Histopathologie Animale, ONIRIS, Ecole Nationale Vétrinaire, Agroalimentaire et de l’Alimentation Nantes-Atlantique, Nantes, France; ^5^LUNAM University, Oniris, AMaROC, Nantes, France; ^6^Broad Institute of MIT and Harvard, Cambridge, MA, 02142, USA; ^7^Science for Life Laboratory, Department of Medical Biochemistry and Microbiology, Uppsala University, Uppsala, 751 23, Sweden

###### **Correspondence:** Céline Le Béguec

Recent efforts have extended the dog genome annotation with the discovery of thousands of long non coding RNAs (lncRNAs) using the machine-learning based tool FEELnc [1]. Although lncRNAs have been shown to play important roles in many biological processes, and particularly in cancers [2], it remains challenging to assign functions and classify lncRNAs in order to interpret their impact on cancers and genetic diseases. Here, we integrated genomic and transcriptomic features from the extended canFam3.1-plus annotation to perform bioinformatic functional predictions of lncRNAs. We first characterized expression patterns of 10,444 canine lncRNAs in 26 distinct tissues representing various histological and anatomical localizations. We defined tissue specificity profiles of lncRNAs and deduced potential functionality and evolutionary origins through comparative genomic and transcriptomic analysis with human data from the ENCODE project (ENCyclopedia Of DNA Elements).

As in human, we show that canine lncRNAs are lower expressed than protein coding genes (mRNAs). Among the 26 tissues, we detected 4,600 tissue-specific lncRNAs. Unsupervised hierarchical clustering based on lncRNA expression levels recapitulates tissue origins and pinpoint candidate lncRNAs likely associated with specialized functions, such as nervous and integumentary clusters. Furthermore, we identified more than 900 conserved dog-human lncRNAs for which we show their overall reproducible expression patterns in both species through comparative transcriptomics. We then constructed co-expression networks and found significant correlations (| rho | > 0.5 and adjusted p-value (BH) < 0.05) for 7,615 lincRNA:mRNA and 524 antisense:mRNA pairs. These results revealed co-expressed modules that may predict regulatory relationships and/or the evolutionary origin of subsets of lncRNAs. Using functional annotations based on GO biological processes terms, we found 23 clusters significantly enriched (adjusted p-value (BH) < 0.05) corresponding to developmental processes ‘sensory organ development’, ‘axon development’ or ‘hindbrain development’.

We conducted a pilot study of melanoma in dogs, we performed differential expression analysis using matched tumour/control RNA-seq samples from canine buccal melanomas. We identified 930 lncRNAs with significant differential expression between tumour and control samples (FDR < 0.01). These lncRNAs represent potential biomarkers and/or candidate to study tumorigenesis of melanomas in dogs. Moreover, more than 100 of the 930 lncRNAs are conserved in human and can be used for further evaluating their therapeutic potential in both human and veterinary medicine. Altogether, this genomic and transcriptomic integrative study of lncRNAs constitutes a major resource for biomedical research in the dog species.

**References**

1. Wucher V. et al. FEELnc: a tool for long non-coding RNA annotation and its application to the dog transcriptome. Nucleic Acids Research. 2017.
2. Gillard M. et al. Naturally occurring melanomas in dogs as models for non-UV pathways of human melanomas. Pigment Cell & Melanoma Research. 2014.

Keywords: Canine, Genomics and Variation, Inherited Disease

### P6 Data cleaning; is it time to stop sweeping it under the carpet? An example from the Dogslife project

#### Charlotte S. C. Woolley, Ian G. Handel, B. Mark Bronsvoort, Jeffrey J. Schoenebeck, Dylan N. Clements

##### The Roslin Institute and the Royal (Dick) School of Veterinary Studies, University of Edinburgh, Edinburgh, UK

###### **Correspondence:** Charlotte S. C. Woolley

Even with careful study design and extensive validation, large datasets are often heterogeneous and require cleaning prior to analysis to prevent losses in research validity, quality and statistical power. Many publications report that data was ‘cleaned’ but few studies document the process reproducibly and values identified as ‘outliers’ are commonly deleted without reporting the possible causes of error. Our aim was to develop a novel, automated data cleaning algorithm for growth (height and weight) that could be applied to large datasets.

Dogslife is an internet-based, longitudinal cohort study of Kennel Club registered Labrador Retrievers living in the UK, which was launched in 2010 and has over 7500 registered dogs to date. The main objective of Dogslife is to identify risk factors for canine health and disease by collecting information from owners via regular questionnaires. In addition to questionnaire data, the study has collected DNA and faecal samples from subsets of the cohort, which has produced genomic and microbiome data.

We developed our data cleaning pipeline in R software and used rule-based approaches, non-linear mixed-effects mathematical models and text analysis to identify common errors such as duplicate entries, typing, decimal point, unit, menu/option, intentional, website-generated and measurement errors. Individuals were permitted to differ from the population by making use of repeated measurements and alternative data sources. The method avoids the modification of unusual but biologically plausible values, prioritise data repair over removal and explicitly report the decision making process behind why a particular data entry is modified or deleted.

We validated our cleaning algorithm for growth variables (weight and height) on three other independent data sources from studies with fundamentally different designs; veterinary consultation Labrador Retriever weight records from the SAVSNET (Small Animal Veterinary Surveillance Network), clinical Labrador Retriever weight records from a veterinary hospital network and a publically available (via the UK Data Service) human weight and height data from CLOSER (Cohort & Longitudinal Studies Enhancement Resources) with varying proportions of artificially simulated errors. We found that our algorithm could be reproducibly applied as an effective data cleaning method on all of the validation datasets. We also compared our method to uncleaned data and six different cleaning methods and found that our algorithm out-performed these with greater accuracy and fewer unnecessary data deletions.

There is an increasing demand for data cleaning methodologies to be thoroughly reported so that they can be reproduced, tested and adapted by the wider research community. In the future, it is vital that data cleaning is considered an integral part of study design and should be considered as early as possible in order to ensure that the quality of the data is conserved. Our methods have broad applicability to longitudinal and cross-sectional growth data and we propose that they could be adapted for use in other breeds, species and fields.

Keywords: Canine, Morphology, Technical Advances

